# Effects of prenatal exercise on gestational weight gain, obstetric and neonatal outcomes: FitMum randomized controlled trial

**DOI:** 10.1186/s12884-023-05507-7

**Published:** 2023-03-29

**Authors:** Caroline B. Roland, Signe dP. Knudsen, Saud A. Alomairah, Anne D. Jessen, Ida K. B. Jensen, Nina Brændstrup, Stig Molsted, Andreas K. Jensen, Bente Stallknecht, Jane M. Bendix, Tine D. Clausen, Ellen Løkkegaard

**Affiliations:** 1 Department of Biomedical Sciences, University of Copenhagen, Copenhagen, Denmark; 2 Department of Gynaecology and Obstetrics, Copenhagen University Hospital – North Zealand, Hilleroed, Denmark; 3 College of Health Sciences, Public Health Department, Saudi Electronic University, Riyadh, Saudi Arabia; 4 Department of Clinical Research, Copenhagen University Hospital – North Zealand, Hilleroed, Denmark; 5 Department of Clinical Medicine, University of Copenhagen, Copenhagen, Denmark; 6 Biostatistics, Department of Public Health, University of Copenhagen, Copenhagen, Denmark

**Keywords:** Maternal exercise interventions, Pregnancy, Physical activity, Gestational weight gain, Delivery, Obstetric and neonatal outcomes

## Abstract

**Background:**

To investigate the effects of two different exercise interventions during pregnancy on gestational weight gain (GWG) and obstetric and neonatal outcomes compared to standard care. Additionally, we aimed to improve standardization of GWG measurements by developing a model to estimate GWG for a standardized pregnancy period of 40 weeks and 0 days accounting for individual differences in gestational age (GA) at delivery.

**Methods:**

In a randomized controlled trial we compared the effects of structured supervised exercise training (EXE) three times per week throughout pregnancy versus motivational counselling on physical activity (MOT) seven times during pregnancy with standard care (CON) on GWG and obstetric and neonatal outcomes. Uniquely, to estimate GWG for a standardized pregnancy period, we developed a novel model to predict GWG based on longitudinally observed body weights during pregnancy and at admission for delivery. Observed weights were fitted to a mixed effects model that was used to predict maternal body weight and estimate GWG at different gestational ages. Obstetric and neonatal outcomes, among them gestational diabetes mellitus (GDM) and birth weight, were obtained after delivery. GWG and the investigated obstetric and neonatal outcomes are secondary outcomes of the randomized controlled trial, which might be underpowered to detect intervention effects on these outcomes.

**Results:**

From 2018–2020, 219 healthy, inactive pregnant women with median pre-pregnancy BMI of 24.1 (21.8–28.7) kg/m^2^ were included at median GA 12.9 (9.4–13.9) weeks and randomized to EXE (*n* = 87), MOT (*n* = 87) or CON (*n* = 45). In total 178 (81%) completed the study. GWG at GA 40 weeks and 0 days did not differ between groups (CON: 14.9 kg [95% CI, 13.6;16.1]; EXE: 15.7 kg [14.7;16.7]; MOT: 15.0 kg [13.6;16.4], *p* = 0.538), neither did obstetric nor neonatal outcomes. For example, there were no differences between groups in the proportions of participants developing GDM (CON: 6%, EXE: 7%, MOT: 7%, *p* = 1.000) or in birth weight (CON: 3630 (3024–3899), EXE: 3768 (3410–4069), MOT: 3665 (3266–3880), *p* = 0.083).

**Conclusions:**

Neither structured supervised exercise training nor motivational counselling on physical activity during pregnancy affected GWG or obstetric and neonatal outcomes compared to standard care.

**Trial registration:**

ClinicalTrials.gov; NCT03679130; 20/09/2018.

**Supplementary Information:**

The online version contains supplementary material available at 10.1186/s12884-023-05507-7.

## Background

It has been suggested that prenatal maternal exercise reduces gestational weight gain (GWG) [1–5] and incidence of other pregnancy and delivery-related complications, including gestational diabetes mellitus (GDM), preeclampsia, gestational hypertension, preterm delivery, caesarean section and odds of instrumental delivery [4, 6–8]. Further, prenatal exercise can reduce duration of labor in some pregnant populations [9] and has been associated with optimization of offspring birth weight into a healthy range [7, 10, 11].

Yet, it remains to be investigated which maternal exercise approaches are most effective for improving health of the mother and her offspring [6]. Structured supervised exercise training and motivational counselling on physical activity constitute two exercise interventions widely used [3, 12]. Both approaches have been applied separately in pregnant women with normal weight [13–19] and overweight or obesity [20–27], but a direct comparison of the effectiveness on improving GWG and obstetric and neonatal outcomes has not been conducted.

The Institute of Medicine has presented ideal and practical methods for measurement and acquisition of body weight data required to determine GWG [28]. Ideally, body weights used to calculate GWG should be pre-pregnancy weight measured at a preconceptional visit and the last measured available weight abstracted from clinical records (ideally at delivery). If not practically feasible to measure maternal weight before conception and at delivery, pre-pregnancy weight and last available weight can be recalled (self-reported) by the women as soon as possible, for example at the first prenatal visit and after delivery, using standardized questions [28]. However, such data will most likely be less precise than objectively measured weights during hospital visits. For practical reasons, most studies calculate GWG based on a weight measured at the last pregnancy visit to the clinic [2, 3, 29], which may vary significantly both between women within a study and between studies, and only few studies have measured weight at delivery and hence reported GWG for the entire pregnancy period [30, 31]. Standardization of GWG measurements at specific gestational age (GA) timepoints during pregnancy can potentially improve comparison of GWG effect sizes between studies.

In this paper, which reports prespecified secondary outcomes of a randomized controlled trial [32], we aimed to investigate the effects of structured supervised exercise training (EXE) or motivational counselling on physical activity (MOT) during pregnancy on GWG and obstetric and neonatal outcomes compared to standard care (CON). Obstetric outcomes included GDM, gestational hypertensive disorders, induction of labor, epidural analgesia, oxytocin augmentation, duration of labor, mode of delivery, rupture degree 3 and 4, and postpartum haemorrhage. Neonatal outcomes included GA at delivery, premature delivery (GA < 37 + 0 weeks), birth weight, birth length, birth weight z-score, small for gestational age (SGA), large for gestational age (LGA), and Apgar score (5 min). Additionally, we aimed to improve standardization of GWG measurements by developing a new model to estimate GWG at specific timepoints during pregnancy and for the entire pregnancy period and account for missing weight measurements during pregnancy as well as individual differences in GA at delivery. Our hypotheses were that GWG would be lower in EXE compared to MOT, and in MOT compared to CON. The remaining investigations in this paper were explorative.

## Methods

### Participants and study procedures

The FitMum study was a randomized controlled trial conducted in 2018–2021 at Copenhagen University Hospital – North Zealand, Hilleroed, Denmark. The study design is described in detail elsewhere [32]. Healthy (without pre-existing or ongoing obstetric or medical complications), inactive (structured exercise at moderate-to-vigorous intensity < 1 h/week during early pregnancy) women with GA ≤ 15 + 0 weeks were eligible for inclusion. The primary objective was to investigate the effect of the two different exercise interventions (EXE and MOT) on moderate-to-vigorous-intensity physical activity during pregnancy compared to CON [33], whereas this paper reports secondary outcomes of the study. Demographic information was obtained at inclusion and pre-pregnancy BMI (kg/m^2^) was calculated based on self-reported pre-pregnancy weight and height. Physical activity, including moderate-to-vigorous-intensity physical activity (min per week), steps (per day), and active kilocalories (per day), was measured continuously from inclusion to delivery by a wrist-worn activity tracker (Garmin Vivosport). Randomization (*n* = 219) in a 1:2:2 pattern to either CON, EXE, or MOT, respectively, occurred after a one-week baseline period (GA ≤ 16 + 0 weeks). Interventions ran from randomization until delivery. Participants in the EXE intervention were offered one-hour supervised exercise training at moderate intensity three times per week, including two exercise sessions in a gym and one in a swimming pool. The gym sessions consisted of a combination of aerobic and resistance training with 30 min stationary bike training and 30 min of other exercise, for example, using elastic bands. In the swimming pool, participants did 15 min of swimming and 45 min of water exercises with plates, balls etc. The MOT intervention consisted of four individual and three group physical activity motivational counselling sessions of 1–2 h duration during pregnancy and a personalized text message once weekly to motivate to increased physical activity. EXE and MOT sessions were conducted by instructors with a bachelor’s or master’s degree in physiotherapy, exercise physiology or similar. During the COVID-19 pandemic, starting from March 11^th^, 2020, and throughout the intervention period, most exercise training sessions, motivational counselling sessions and periodically test visits (except delivery) were conducted online from home using Zoom Cloud Meetings or telephone. EXE could access to the swimming pool for three months during this period.

### Outcome measurements

#### Gestational weight gain

Pre-pregnancy body weight was self-reported by the participants. From inclusion, all weight measurements were recorded to the nearest 0.1 kg on calibrated electronic scales (SECA799) at baseline (GA ≤ 15 + 0 weeks), GA 28 + 0–6 and 34 + 0–6 weeks (visit 2 and 3), and at delivery. During COVID-19, women were weighed at home on private scales and weights were self-reported. To estimate GWG for a standardized pregnancy period of 40 + 0 weeks (from here called *Total GWG*) and account for missing measurements and individual differences in GA at delivery, all observed weights (self-reported and measured) were fitted to a mixed effects model to predict the weights at specific timepoints throughout pregnancy at the participant-level. GWG was estimated at GA 12 + 0, 28 + 0, and 40 + 0 weeks as the difference between the predicted weight and predicted pre-pregnancy weight (GA = 0).

#### Obstetric and neonatal outcomes

Obstetric and neonatal outcomes were collected from medical records. Obstetric outcomes included pregnancy complications (GDM and gestational hypertensive disorders) and delivery-related outcomes (induction of labor, epidural analgesia, oxytocin augmentation, duration of labor, mode of delivery, rupture degree 3 and 4, postpartum haemorrhage). Gestational hypertensive disorders included gestational hypertension, preeclampsia, HELLP syndrome and eclampsia, which were defined based on recommendations from the International Society for the Study of Hypertension in Pregnancy [34] and evaluated by a physician. Total duration of labor included the time from the active phase (starting when cervix was dilated 4 cm and the woman had regular contractions) until the baby was born. The active second stage was defined as the time of active pushing. Neonatal outcomes included GA at delivery, premature delivery (GA < 37 + 0 weeks), birth weight, birth length, birth weight z-score, SGA, LGA and Apgar score (5 min). Birth weight was transformed to a z-score, and SGA (< 10th percentile) and LGA (> 90th percentile) [35] were defined for a Danish standard population and calculated from the Marsal formula [36], which includes fetal sex, birth weight and GA.

### Statistical analysis

A retrospective sample size calculation performed for the prespecified secondary outcome, GWG, showed an estimated sample size of 33 participants in CON and 66 participants in each intervention group to detect a difference of 2.8 kg between CON and the intervention groups (statistical analysis plan available with trial registration at clinicaltrials.gov). Data are presented as mean ± standard deviation (SD) for approximately symmetric distributions, median and interquartile range (IQR) for asymmetric distributions, and frequency and proportion for categorical data. Estimated effect sizes are presented with 95% confidence interval [95% CI]. Statistical analyses were performed using R [37] and statistical significance was defined as a *p*-value below 5%.

Analysis of GWG was based on the intention-to-treat principle including all randomized participants. Trajectories of observed gestational weights during pregnancy were modelled by a mixed effects model featuring an intercept constrained to be equal across groups due to the randomized design [38]. Group-specific change-points were included in the model to allow for a piece-wise linear relationship with two different slopes over time in each of the groups. This led to a total of ten fixed effects in the model consisting of the common intercept and two different slopes and the change-point for each of the three groups. Normal distributed random effects were included at the subject level as intercepts and the two slopes with an unstructured covariance matrix. The model was implemented in Stan [39] and estimated using Markov-Chain Monte Carlo in four parallel chains each running for 10,000 iterations with half of them used for warm-up. A uniform distribution between 50 and 250 days was used as priors for the change-points. The fitted model was subsequently applied to predict individual weights at predetermined timepoints.

We used the randomized controlled trial design to investigate differences between groups in GWG and obstetric and neonatal outcomes, and an observational design combining all participants independent of group allocation to investigate associations between prenatal physical activity measures per se and GWG. Between-group comparisons of estimated GWG after each trimester were performed using analysis of variance (ANOVA). A sensitivity analysis was conducted using linear regression to investigate total GWG in each group before and during COVID-19, where the intervention groups received in-person and online interventions, respectively. Another sensitivity analysis using ANOVA included only participants, whose weight were measured at the hospital, to investigate the influence of weight measurements being obtained by the calibrated scale at the hospital versus via the participants’ home scales. For obstetric and neonatal outcomes, differences between groups were tested with Pearson’s χ^2^ test for categorical variables, ANOVA for symmetrically distributed variables, and Kruskal–Wallis test for asymmetrically distributed variables. Post-hoc pairwise comparisons were performed using Pearson’s χ^2^ test with Holm-corrected *p*-values, Tukey’s method, or Wilcoxon rank sum test with Holm-corrected *p*-values for categorical, symmetrically distributed, and asymmetrically distributed variables, respectively.

Associations between physical activity measures and total GWG among all participants were performed using linear regression. Physical activity measures (moderate-to-vigorous-intensity physical activity (min per week), steps (per day), and active kilocalories (per day)) used for association analyses were the average values from randomization to delivery day for participants who delivered at GA ≤ 40 + 0 weeks, and from randomization to GA 40 + 0 weeks for participants who were lost to follow-up before delivery or delivered at GA > 40 + 0 weeks.

## Results

**Fig. 1:**
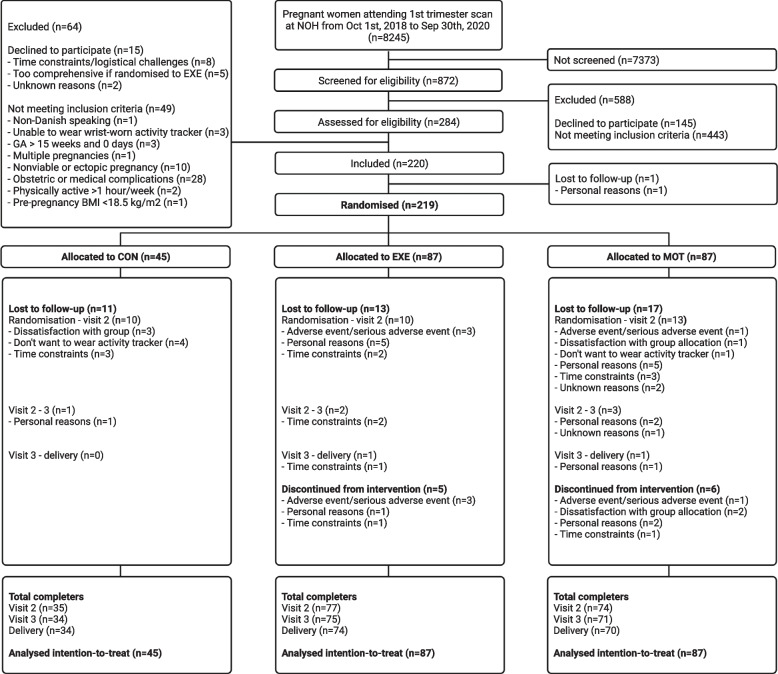
Inclusion, randomization, allocation and completion of the FitMum study reported in accordance with the CONSORT (Consolidated Standards of Reporting Trials) guidelines [40]. CON; Control, EXE; Structured supervised exercise training, MOT; Motivational counselling on physical activity. The figure was created with BioRender.com

We included 220 participants from GA 6 + 1–15 + 0 weeks. One participant was lost to follow-up before randomization and hence, 219 women with median pre-pregnancy BMI of 24.1 (21.8–28.7) kg/m^2^ were randomized to CON (*n* = 45), EXE (*n* = 87) and MOT (*n* = 87) (Fig. 1). Maternal baseline characteristics are presented in Table 1. All 219 participants were included in the analysis of GWG. From randomization to delivery, 19% of the participants were lost to follow-up, thus data from 178 participants (CON: *n* = 34; EXE: *n* = 74; MOT: *n*= 70) were included in the analyses of obstetric and neonatal outcomes. Lost to follow-up rate did not differ between groups. Adverse and serious adverse events did not differ between groups (data not shown) and the interventions did not seem to negatively influence the mother or offspring. The average weekly moderate-to-vigorous-intensity physical activity from randomization to delivery was 35.4 min [19.4;51.4] in CON, 53.5 min [42.0;65.0] in EXE, and 43.1 min [31.6;54.6] in MOT. The average daily steps from randomization to delivery was 6896 [6408;7383] in CON, 6680 [6331;7028] in EXE, and 6792 [6441;7143] in MOT. The average daily active kilocalories from randomization to delivery was 562 [504;619] in CON, 560 [518;601] in EXE, and 583 [541;625] in MOT [33]. Adherence to the interventions in EXE and MOT was on average 1.3 [1.1;1.5] out of 3 sessions per week and 5.2 [4.7;5.7] out of 7 pregnancy counselling sessions, respectively [33].

**Table 1:** Maternal baseline characteristics

**Characteristic**	**ALL**	**CON**	**EXE**	**MOT**
***Number of participants***	***n* = *219***	***n* = *45***	***n* = *87***	***n* = *87***
Age, years	31.5 ± 4.3	32.0 ± 4.6	31.1 ± 4.3	31.7 ± 4.1
Pre-pregnancy BMI, kg/m^2a^	24.1 (21.8–28.7)	23.5 (21.3–26.8)	25.2 (21.6–29.8)	24.1 (22.4–28.9)
Gestational age, weeks (median)	12.9 (9.4–13.9)	12.9 (9.7–13.9)	12.6 (9.3–13.7)	12.9 (9.6–13.9)
Gestational age, weeks (mean)	11.7 ± 2.5	11.9 ± 2.6	11.7 ± 2.4	11.8 ± 2.4
Parity, nulliparous, n (%)	82 (37%)	16 (36%)	40 (46%)	26 (30%)
Education level, n (%)^b^				
School ≥ 12 years	191 (87%)	41 (91%)	74 (85%)	76 (87%)
Further education ≥ 3 years	175 (80%)	33 (73%)	73 (84%)	69 (79%)
Employed/studying, n (%)	199 (91%)	39 (87%)	83 (95%)	77 (89%)
Smoking, n (%)				
During pregnancy	2 (1%)	0 (0%)	1 (1%)	1 (1%)
Quit smoking before pregnancy	27 (12%)	6 (13%)	12 (14%)	9 (10%)

Baseline characteristics in the randomization groups

Data are mean ± SD, median (IQR) and n (%). No statistical comparisons have been performed on baseline characteristics in accordance with CONSORT recommendations

*CON* Control, *EXE* Structured supervised exercise training, *MOT* Motivational counselling on physical activity, *BMI* Body mass index, *SD* Standard deviation, *IQR* Interquartile range

^a^ Pre-pregnancy BMI; *n* = 218 (CON; *n* = 45, EXE; *n* = 86, MOT; *n* = 87)

^b^ School ≥ 12 years corresponds to high school, and further education ≥ 3 years corresponds to university degree (bachelor or master level)

### Gestational weight gain after each trimester

**Fig. 2:**
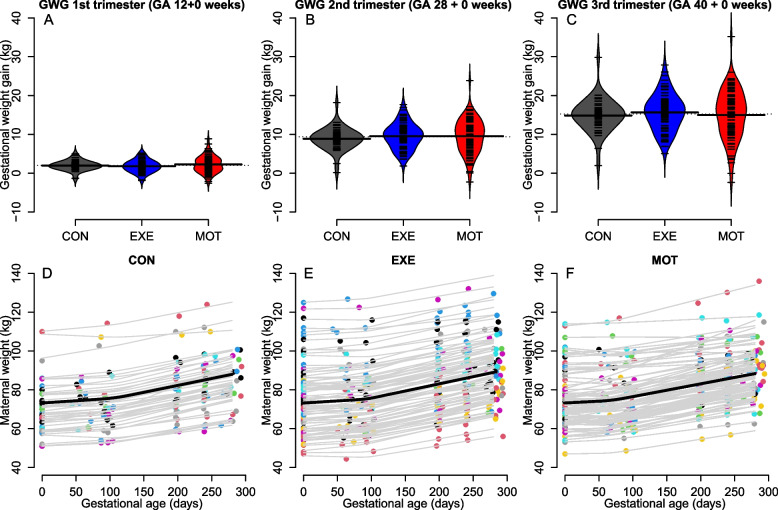
Gestational weight gain (GWG) for all participants (*n* = 219) after first trimester/GA 12 + 0 weeks (**A**), second trimester/GA 28 + 0 weeks (**B**), and third trimester/GA 40 + 0 weeks (total GWG) (**C**). Self-reported and measured weights (dots) and predicted weights by mixed effects model (lines) for all individuals throughout pregnancy in the three groups (**D-F**). ANOVA was used for **A-C** and showed no differences between groups at GA 12 + 0 weeks (*p* = 0.310), GA 28 + 0 weeks (*p* = 0.396) and GA 40 + 0 weeks (*p* = 0.538). CON; Control, EXE; Structured supervised exercise training, MOT; Motivational counselling on physical activity, GWG; Gestational weight gain, GA; Gestational age

Estimated GWG did not differ between groups at GA 12 + 0 weeks (*p* = 0.310) or GA 28 + 0 weeks (*p* = 0.396) (Fig. 2A-B). Total GWG (GWG at GA 40 + 0 weeks) was 14.9 kg [13.6;16.1] in CON, 15.7 kg [14.7;16.7] in EXE and 15.0 kg [13.6;16.4] in MOT and did not differ between groups (*p* = 0.538) (Fig. 2C). Pairwise comparisons of total GWG showed no differences in total GWG between either MOT and EXE (-0.7 kg [-2.6;1.3], *p* = 0.710), MOT and CON (0.2 kg [-2.0;2.3], *p* = 0.985), or EXE and CON (0.8 kg [-1.1;2.7], *p* = 0.562). Figure 2D-F illustrates the estimated relationships between self-reported and measured body weight observations (dots) and predicted body weights by the mixed effects model (lines) for all individuals in the three groups. A complete case analysis of GWG calculated traditionally as weight measured at delivery (available for *n* = 131) minus self-reported pre-pregnancy weight showed no difference between groups (*p* = 0.612) (Figure S.1). Sensitivity analyses showed higher total GWG (4.7 kg [1.6;7.8], *p* = 0.003) in MOT during COVID-19 compared with before COVID-19, but no differences were found in EXE (1.6 kg [-0.8;4.0], *p* = 0.184) and CON (-1.2 kg [-4.3;1.9], *p* = 0.425). We found no differences between the three study groups in total GWG among participants, whose weights were measured at the hospital only (*n* = 167, *p* = 0.537).

### Associations between prenatal physical activity per se and GWG

We investigated if physical activity per se was associated with total GWG independently of group allocation. We found no associations between any of the physical activity measures and total GWG (moderate-to-vigorous-intensity physical activity: slope -0.01 [-0.02;0.01], *p* = 0.363; steps: slope 0.14∙10^–3^ [-0.03∙10^–2^;0.06∙10^–2^], *p* = 0.537; active kilocalories: slope -0.09∙10^–2^ [-0.05∙10^–1^;0.03∙10^–1^], *p* = 0.637) (Figure S.2A-C).

### Obstetric and neonatal outcomes

Overall, none of the obstetric and neonatal outcomes differed between groups (Tables 2 and 3) apart from GA at delivery (*p* = 0.048). Holm-corrected post-hoc pairwise comparisons showed that EXE had higher GA at delivery compared to MOT (EXE: 40.6 weeks (39.9–41.3), MOT: 40.0 weeks (39.3–40.9), *p* = 0.038) (Table 3).

**Table 2:** Obstetric outcomes

**Outcome**	**ALL**	**CON**	**EXE**	**MOT**	***P*-value**
***Number of participants***	***n* = *178***	***n* = *34***	***n* = *74***	***n* = *70***	
***Pregnancy complications***					
GDM, n (%)	12 (7%)	2 (6%)	5 (7%)	5 (7%)	1.000^a^
Gestational hypertensive disorders, n (%)*	11 (6%)	2 (6%)	4 (5%)	5 (7%)	0.919^a^
***Delivery related outcomes***					
Induction of labor, n (%)	53 (30%)	11 (32%)	22 (30%)	20 (29%)	0.925^a^
Mode of delivery, n (%)					
Unassisted vaginal	138 (78%)	30 (88%)	60 (81%)	48 (69%)	0.050^a^
Instrumental assisted vaginal	8 (5%)	1 (3%)	1 (1%)	6 (9%)	0.103^a^
Planned caesarean section	11 (6%)	1 (3%)	5 (7%)	5 (7%)	0.791^a^
Emergency caesarean section	21 (12%)	2 (6%)	8 (11%)	11 (16%)	0.333^a^
Epidural analgesia, n (%)	58 (33%)	9 (27%)	25 (34%)	24 (34%)	0.698^a^
Oxytocin augmentation, n (%)	46 (26%)	5 (15%)	24 (32%)	17 (24%)	0.138^a^
Rupture degree 3 + 4, n (%)**	8 (5%)	1 (3%)	3 (4%)	4 (6%)	0.805^a^
Postpartum haemorrhage, ml	350 (250–508)	300 (200–445)	350 (250–593)	400 (250–540)	0.212^b^
Postpartum haemorrhage > 1000 ml, n (%)	19 (11%)	2 (6%)	11 (15%)	6 (9%)	0.300^a^
Duration of labor nulliparous					
Total duration vaginal delivery, min**	443 (273–617)	523 (199–582)	481 (363–678)	298 (198–488)	0.163^b^
Duration of active second stage labor, min**	41 (22–65)	53 (16–70)	43 (23–67)	34 (21–44)	0.436^b^
Active second stage labor > 60 min, n (%)**	15 (27%)	3 (27%)	9 (31%)	3 (20%)	0.797^a^
Duration of labor multiparous					
Total duration vaginal delivery, min**	150 (86–262)	126 (102–254)	152 (79–277)	160 (88–262)	0.994^b^
Duration of active second stage labor, min**	13 (7–19)	13 (6–20)	14 (7–19)	11 (6–19)	0.849^b^
Active second stage labor > 30 min, n (%)**	11 (13%)	3 (16%)	2 (7%)	6 (16%)	0.592^a^

Obstetric outcomes in the randomization groups reported for participants still enrolled in the study at delivery (*n* = 178)

Data are mean ± SD, median (IQR) and n (%)

*CON* Control, *EXE* Structured supervised exercise training, *MOT* Motivational counselling on physical activity, *GDM* Gestational diabetes mellitus, *SD* Standard deviation, *IQR* Interquartile range

^*^ Defined as preeclampsia, gestational hypertension, HELLP or eclampsia

^**^ For some variables the total number is lower due to missing values: For nulliparous women: Total duration of vaginal deliveries; *n* = 49 (CON; *n* = 10, EXE; *n* = 24, MOT; *n* = 15), duration of active second stage labor; *n* = 55 (CON; *n* = 11, EXE; *n* = 29, MOT; *n* = 15), active second stage labor > 60 min; *n* = 55 (CON; *n* = 11, EXE; *n* = 29, MOT; *n* = 15), for multiparous women: total duration of vaginal deliveries; *n* = 81 (CON; *n* = 19, EXE; *n* = 27, MOT; *n* = 35), duration of active second stage labor; *n* = 86 (CON; *n* = 19, EXE; *n* = 29, MOT; *n* = 38), active second stage labor > 30 min; *n* = 86 (CON; *n* = 19, EXE; *n* = 29, MOT; *n* = 38), rupture degree 3 + 4; *n* = 173 (CON; *n* = 31, EXE; *n* = 72, MOT; *n* = 70)

^a^Pearson’s χ^2^ test, ^b^Kruskal-Wallis test

**Table 3:** Neonatal outcomes

**Outcome**	**ALL**	**CON**	**EXE**	**MOT**	***P*-value**
***Number of participants***	***n* = *178***	***n* = *34***	***n* = *74***	***n* = *70***	
Gestational age delivery, weeks (median)	40.4 (39.4–41.1)	40.2 (38.8–41.3)	40.6 (39.9–41.3)	40.0 (39.3–40.9)	0.048^b#^
Gestational age delivery, weeks (mean)	40.1 ± 1.6	39.8 ± 1.9	40.4 ± 1.2	39.8 ± 1.7	
Premature delivery (GA < 34), n (%)	3 (2%)	1 (3%)	0 (0%)	2 (3%)	0.309^a^
Premature delivery (GA 34 + 0–36 + 6), n (%)	3 (2%)	2 (6%)	1 (1%)	0 (0%)	0.093^a^
Birth weight, g	3715 (3289–3979)	3630 (3024–3899)	3768 (3410–4069)	3665 (3266–3880)	0.083^b^
Birth length, cm*	52 (51–53)	52 (51–54)	53 (51–54)	52 (51–53)	0.354^b^
Birth weight adjusted for GA at delivery and sex, z-score	0.10 ± 1.0	-0.02 ± 1.0	0.17 ± 1.0	0.09 ± 1.0	0.648^c^
SGA (< 10th percentile), n (%)	15 (8%)	4 (12%)	3 (4%)	8 (11%)	0.208^a^
LGA (> 90th percentile), n (%)	18 (10%)	2 (6%)	7 (10%)	9 (13%)	0.526^a^
5-min apgar score < 7, n (%)	1 (1%)	0 (0%)	0 (0%)	1 (1%)	0.580^a^

Neonatal outcomes in the randomization groups reported for participants still enrolled in the study at delivery (*n* = 178)

Data are mean ± SD, median (IQR) and n (%)

*CON* Control, *EXE* Structured supervised exercise training, *MOT* Motivational counselling on physical activity, *GA* Gestational age, *SGA* Small for gestational age infants, *LGA* Large for gestational age infants, *SD* Standard deviation, *IQR* Interquartile range

^*^ Birth length; *n* = 177 (CON; *n* = 34, EXE; *n* = 74, MOT; *n* = 69)

^a^Pearson’s χ^2^ test, ^b^Kruskal-Wallis test, ^c^Analysis of variance (ANOVA). ^#^EXE vs. MOT (*p* = 0.038) (pairwise Wilcoxon rank sum test with Holm-corrected *p*-value)

## Discussion

We found no effect of either EXE or MOT during pregnancy on GWG and obstetric and neonatal outcomes in healthy pregnant women compared to CON. Hence, our predefined hypotheses that GWG would be lower in EXE compared to MOT, and in MOT compared to CON were rejected. Overall, the GWG and incidences of obstetric and neonatal outcomes were within the Institute of Medicine’s recommendations for pregnant women with normal weight [28] and corresponded to the general incidences in Denmark [41, 42], respectively. Notably, the interventions did not seem to influence the mother or offspring negatively, which is in line with previous studies [43].

It is noteworthy that the interventions did not reduce GWG compared to CON. These findings contrast with several studies showing reduced GWG compared to standard care after prenatal exercise in healthy [3] and normal weight women [44]. However, some studies also found no effect of exercise on GWG in women with normal weight [19, 45]. Moreover, our findings of no effects of the interventions on obstetric and neonatal outcomes contradict most studies reporting a protective effect of prenatal exercise on GDM and hypertensive disorders [3] and reduced preterm delivery, SGA and LGA [11] in women with normal weight, overweight and obesity. However, similar to the present study, other studies found no effects of prenatal exercise on GDM, preeclampsia, preterm delivery and birth weight [45–47]. The literature is inconsistent regarding the effects of prenatal exercise on mode of delivery, induction of labor and use of epidural analgesia [6, 48, 49]. Given the importance of achieving a certain amount of physical activity to obtain beneficial effects [50, 51], the lack of effect from interventions in the present study might be explained by a low adherence rate and low moderate-to-vigorous-intensity physical activity level among participants in EXE and MOT. In both EXE and MOT the moderate-to-vigorous-intensity physical activity level was below one hour per week and thus markedly lower than the recommended physical activity level by the Danish Health Authorities of 210 min per week at moderate intensity [51]. COVID-19 did not seem to affect physical activity level negatively in our study. Moderate-to-vigorous-intensity physical activity did not differ between participants included before the COVID-19 pandemic (physical intervention only) and during the COVID-19 pandemic (online intervention only) in any of the three study groups [33]. The online intervention attendance rate in EXE during COVID-19 was significantly higher (women participating in more exercise sessions per week) compared to the physical EXE intervention [33].

However, even if assuming that our interventions would be effective on reducing GWG and improving obstetric and neonatal outcomes had adherence rate and moderate-to-vigorous-intensity physical activity level been higher, the present study might still be underpowered to detect effects on GWG and the investigated obstetric and neonatal outcomes. Despite that we fulfilled the sample size estimated from our sample size calculation for GWG, this calculation might have been based on too optimistic estimates [17] compared to effect sizes of lifestyle and physical activity interventions on GWG generally reported in the literature [1, 4, 6, 44].

Another possible explanation of the negative findings on health outcomes in the present study could be that the results might be biased by the Hawthorne effect since it is uncertain to what extent CON interacted with the activity tracker and improved physical activity level as a result hereof. This could challenge detection of possible differences between intervention groups and CON. However, none of the physical activity measures were per se associated with GWG.

Regarding health status of our overall study population, the prevalence of overweight or obesity [52] and the level of insufficient PA [53] are lower among Danish women in general compared to women in other western countries such as the United States and the United Kingdom. This might reduce the potential for exercise to induce beneficial health effects on GWG and obstetric and neonatal outcomes in our study population compared to pregnant populations in other countries with a higher overweight and obesity burden among pregnant women, for example the United States [54]. Thus, intervention effects of EXE and MOT on reduced GWG and improved obstetric and neonatal outcomes would probably have been more evident among populations with overweight or obesity.

The GWG data included in this paper do not take the maternal body composition into account. Since exercise-induced improvement of body composition has been shown in both pregnant [55] and non-pregnant populations [56, 57], further investigation on whether maternal body composition might be improved in EXE or MOT compared to CON is needed. Furthermore, a limitation of the study is that dietary intake was not monitored. Diet interventions have been found to result in lower GWG than physical activity interventions when compared to control [4]. Thus, at least intervening on dietary intake seems to influence GWG more than physical activity interventions, and possible differences in dietary intake in our study might have blurred potential effects of exercise.

A strength of the present study is that the last weight measurement is obtained at delivery enabling us to develop a novel model to estimate GWG for a standardized pregnancy period of 40 + 0 weeks accounting for missing weight measurements and individual differences in GA at delivery. Using this model, we showed a good fit between observed weights and model-predicted weights for all individuals in all three groups, and a complete-case analysis including only participants with weight measurements at delivery showed similar results of GWG as the results of estimated GWG by the model. Thus, this novel method can be used to precisely estimate GWG at specific timepoints throughout pregnancy, for example GWG for the entire pregnancy period. Further, this method allowed us to take GA into account, which can vary up to five weeks within term-deliveries and thus likely influence GWG markedly. Moreover, missing data could be predicted by the model allowing us to report mean GWG at GA 40 + 0 weeks for all 219 study participants even though we only obtained weight data on 131 women at delivery. Thus, this model can be used to standardize measurements of GWG at specific GA timepoints during pregnancy, which can improve comparison of GWG effect sizes between studies. Including self-reported pre-pregnancy weights in the model constitutes a limitation but is justified by the finding that the weight gain slopes are visually comparable between participants in all three groups with no significant outliers.

## Conclusions

The present randomized controlled study compared two different exercise intervention strategies during pregnancy and found no effect of either structured supervised exercise training or motivational counselling on physical activity on GWG and obstetric and neonatal outcomes compared to standard care. The study proposes a novel method to estimate GWG for a standardized pregnancy period of 40 + 0 weeks, which may contribute to advance state-of-the-art in the obstetric research field.

## Supplementary Information

**Additional file 1: Figure S.1.** Complete case analysis of gestational weight gain at delivery including participants with available weight measurements from delivery only (*n*=131). ANOVA showed no difference between groups (*p*=0.612). CON; Control, EXE; Structured supervised exercise training, MOT; Motivational counselling on physical activity.

**Additional file 2: Figure S.2.** Associations between moderate-to-vigorous-intensity physical activity (min/week) and total gestational weight gain (GWG) (A), steps per day and total GWG (B), and active kilocalories per day and total GWG (C) for all participants (*n*=219). Data points are visualized based on average moderate-to-vigorous-intensity physical activity, steps and active kilocalories of 25 imputed data sets. A linear regression analysis showed no associations between moderate-to-vigorous-intensity physical activity (*p*=0.363), steps(*p*=0.537), active kilocalories (*p*=0.637) and total GWG, respectively. GWG; Gestational weight gain.

## Data Availability

The datasets generated and/or analyzed during the current study are not publicly available due to confidentiality but are available from the corresponding author on reasonable request. We can transfer individual participant data when we have obtained approval from the Danish Data Protection Authority according to the Data Protection Act and completed a SCC (Standard Contractual Clause) to ensure the legal basis of the transfer.

## Abbreviations

BMI: Body mass index
CON: Standard care
EXE: Structured supervised exercise training
GA: Gestational age
GDM: Gestational diabetes mellitus
GWG: Gestational weight gain
IQR: Interquartile range
LGA: Large for gestational age
MOT: Motivational counselling on physical activity
SD: Standard deviation
SGA: Small for gestational age
