# Supplementary material for: Effects of prenatal exercise on gestational weight gain, obstetric and neonatal outcomes: FitMum randomized controlled trial
Source: BMC Pregnancy Childbirth. 2023 Mar 29;23:214. doi: 10.1186/s12884-023-05507-7 (PMC10050797; doi:10.1186/s12884-023-05507-7)

Figure S.1: Complete case analysis of gestational weight gain at delivery including participants with available weight measurements from delivery only (n=131). ANOVA showed no difference between groups (p=0.612). CON: Control, EXE: Structured supervised exercise training, MOT: Motivational counselling on physical activity

Gestational weight gain at delivery

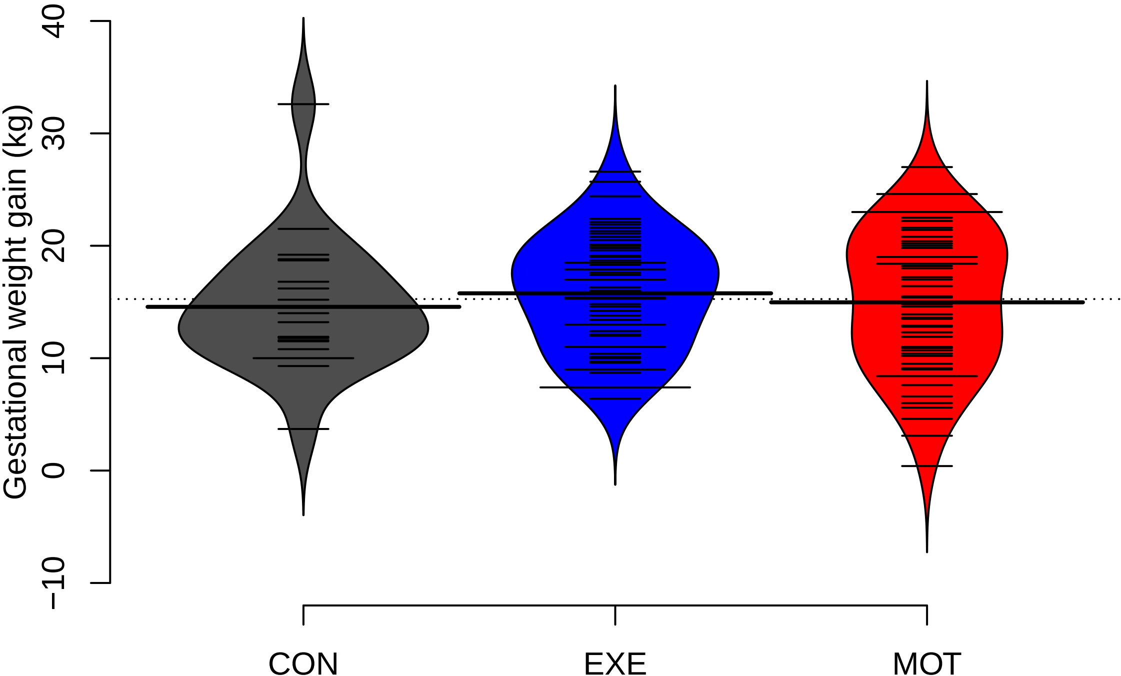

Supplement: Supplementary file 1 — Additional file 1: Figure S.1. Complete case analysis of gestational weight gain at delivery including participants with available weight measurements from delivery only (n=131). ANOVA showed no difference between groups (p=0.612). CON; Control, EXE; Structured supervised exercise training, MOT; Motivational counselling on physical activity. [file 12884_2023_5507_MOESM1_ESM.pdf]
