# Supplementary material for: Effects of prenatal exercise on gestational weight gain, obstetric and neonatal outcomes: FitMum randomized controlled trial
Source: BMC Pregnancy Childbirth. 2023 Mar 29;23:214. doi: 10.1186/s12884-023-05507-7 (PMC10050797; doi:10.1186/s12884-023-05507-7)
Supplement: Supplementary file 2 — Additional file 2: Figure S.2. Associations between moderate-to-vigorous-intensity physical activity (min/week) and total gestational weight gain (GWG) (A), steps per day and total GWG (B), and active kilocalories per day and total GWG (C) for all participants (n=219). Data points are visualized based on average moderate-to-vigorous-intensity physical activity, steps and active kilocalories of 25 imputed data sets. A linear regression analysis showed no associations between moderate-to-vigorous-intensity physical activity (p=0.363), steps(p=0.537), active kilocalories (p=0.637) and total GWG, respectively. GWG; Gestational weight gain. [file 12884_2023_5507_MOESM2_ESM.pdf]

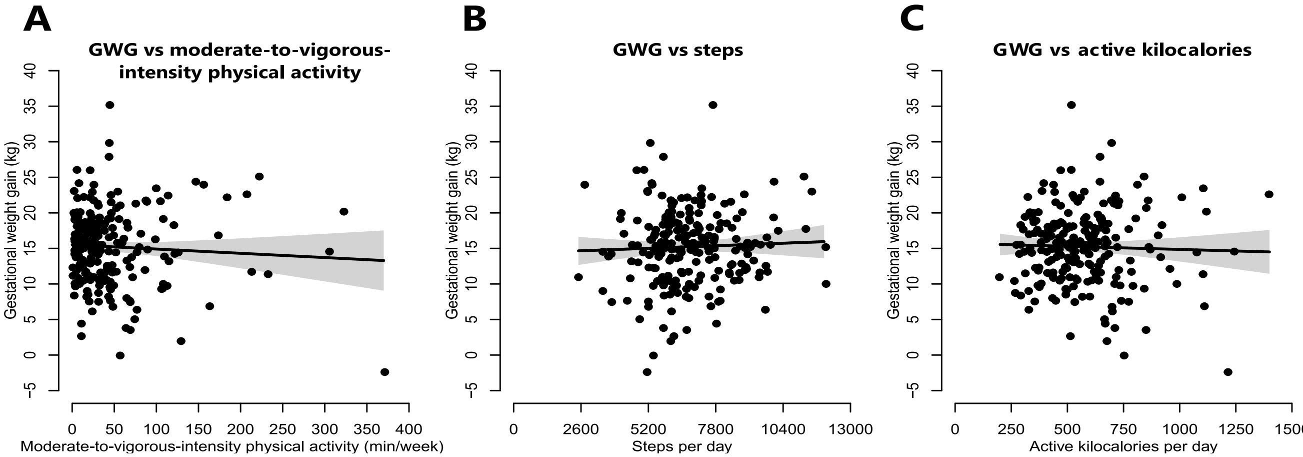

Figure S.2: Associations between moderate-to-vigorous-intensity physical activity (min/week) and total gestational weight gain (GWG) (A), steps per day and total GWG (B), and active kilocalories per day and total GWG (C) for all participants (n=219). Data points are visualized based on average moderate-to-vigorous-intensity physical activity, steps and active kilocalories of 25 imputed data sets. A linear regression analysis showed no associations between moderate-to-vigorous-intensity physical activity ($p=0.363$), steps($p=0.537$), active kilocalories ($p=0.637$) and total GWG, respectively. GWG; Gestational weight gain.
